# An Update on Semen Physiology, Technologies, and Selection Techniques for the Advancement of In Vitro Equine Embryo Production: Section I

**DOI:** 10.3390/ani11113248

**Published:** 2021-11-13

**Authors:** Morgan F. Orsolini, Stuart A. Meyers, Pouya Dini

**Affiliations:** 1 Department of Population Health and Reproduction, School of Veterinary Medicine, University of California, Davis, CA 95616, USA; mforsolini@ucdavis.edu; 2 Department of Anatomy, Physiology, and Cell Biology, School of Veterinary Medicine, University of California, Davis, CA 95616, USA; smeyers@ucdavis.edu

**Keywords:** stallion, fertility, sperm, assisted reproductive techniques

## Abstract

**Simple Summary:**

Male fertility is often estimated by simple sperm assessment, and therefore, it is crucial to establish species-specific baselines for normal sperm parameters. In this paper, sperm physiology, function, and common abnormalities in stallions will be reviewed.

**Abstract:**

As the use of assisted reproductive technologies (ART) and in vitro embryo production (IVP) expand in the equine industry, it has become necessary to further our understanding of semen physiology as it applies to overall fertility. This segment of our two-section review will focus on normal sperm parameters, beginning with development and extending through the basic morphology of mature spermatozoa, as well as common issues with male factor infertility in IVP. Ultimately, the relevance of sperm parameters to overall male factor fertility in equine IVP will be assessed.

## 1. Introduction

During natural breeding, a stallion will deposit millions of sperm within the intra-uterine environment of the mare [1]. Among this population of sperm there is a wide array of sperm “quality”, which represents the ability of the sperm to fertilize an oocyte and produce viable offspring [2]. Although some variation in sperm morphology and physiology between either individuals of the same species or within an ejaculate will not affect fertilization and embryo development outcomes, some parameters are correlated with fertilization, embryo development, and pregnancy outcomes.

This significant diversity in sperm fertility within an ejaculate becomes more pertinent when applied to in vitro embryo production (IVP), during which a smaller number of sperm are generally selected for either in vitro fertilization (IVF) or intracytoplasmic sperm injection (ICSI). Thus, it becomes necessary to understand which physiological and functional parameters are the biggest contributors to sperm fertility. This allows us to select the highest quality sperm within an ejaculate for IVP.

Studies of equine sperm fertility encompass sperm biogenesis [3,4], motility and metabolism [5,6], morphology [7], sperm ultrastructure [8], and biochemical elements of sperm function [9,10,11,12], including sperm interactions with accessory sex gland secretions [13,14,15]. The wholistic picture of sperm fertility is integral to the maximization of IVP outcomes, and, therefore, in Section I of this review we will focus on equine spermatogenesis, sperm morphology, and common sperm abnormalities leading to infertility.

## 2. Spermatogenesis

**Figure 1:**
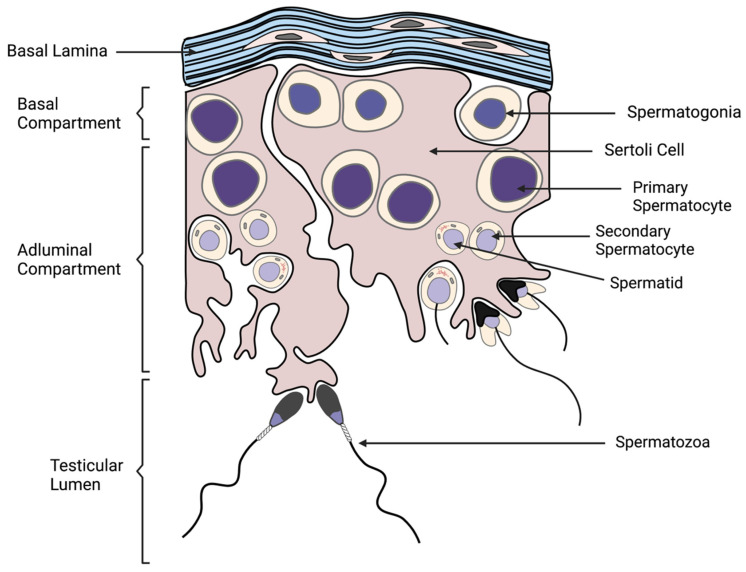
Schematic presentation of spermatogenesis. Facilitated by the nurturing Sertoli cells, basal spermatogonia replicate and differentiate into primary spermatocytes, and sequentially develop into secondary spermatocytes, spermatids, and the morphologically distinct spermatozoa during spermatogenesis. Spermatozoa are released into the lumen of the seminiferous tubule of the testis during spermiation.

Adequate production of high-quality sperm by the male is critical to both natural and artificial reproductive processes. Therefore, it is critical to understand the pathways by which male gametes are derived. This process, known as spermatogenesis, occurs in the germinal epithelium of the seminiferous tubules of the testis, and is initiated during puberty (Figure 1) [3]. Cross sections of the seminiferous tubules reveal adjacent cellular associations that produce sperm in a cyclic manner, repeating approximately every 12 days in the stallion for the constant production of spermatozoa [3,16,17,18].

The seminiferous tubule is divided into a basal and an adluminal layer, which is fully surrounded by a basal lamina [3,19]. Leydig cells, which are stimulated by Luteinizing Hormone (LH) to produce sex hormones, including testosterone, are key for regulating spermatogenesis as well as being responsible for the male phenotype [20,21]. Leydig cells occupy the interstitial space of the testes between adjacent seminiferous tubules and serve as a key regulator of Sertoli cell function [21]. Within the seminiferous tubules, Sertoli cells span both the basal and adluminal layers, and their role is to host germ cells as they undergo meiosis and differentiation [22,23,24]. Specifically, Sertoli cells are stimulated by pituitary Follicle Stimulating Hormone (FSH) and secrete a variety of proteins that play a role in germ cell nourishment and development [22,24]. In the stallion, it has been shown that seasonal fertility is partially attributed to changes in the number of Sertoli cells in the testis, which is directly correlated with the numbers of spermatozoa ultimately produced [25,26].

The process by which mature spermatozoa are generated is a highly regulated process spanning across multiple domains of the testis. A non-committed store of A-spermatogonial cells exists at the basal layer and remains undifferentiated due to the expression of the Neurogenin 3 (*NGN3*) gene [27,28,29]. However, A-spermatogonia exist both to replenish the population of gametic stem cells and differentiate for continuation of spermatogenesis, and, therefore, a subpopulation of A-spermatogonia become committed to differentiation [27,30]. The basal store of cells begin as single cells (A_single_) and undergo either a complete mitotic division forming two single daughter cells, or several rounds of incomplete mitosis in order to create paired (A_paired_) and aligned (A_aligned_) cell groups connected by intercellular bridges [30]. A_aligned_ spermatogonia then undergo differentiation into committed A_1_-spermatogonia, which also reside in the basal compartment [31]. However, A_1_ cells do not express *NGN3* and, therefore, will undergo several rounds of mitosis and differentiation while remaining connected by intercellular bridges (A1, A2, A3, B1, B2 stages) [3,4,27,32]. This period of cell replication is known as spermatocytogenesis and, ultimately, produces preleptotene primary spermatocytes [4]. These primary spermatocytes then enter the meiosis phase, where they pass into the adluminal compartment and participate in two meiotic divisions, first becoming haploid secondary spermatocytes and, ultimately, producing haploid spermatids [3,4].

Following spermatocytogenesis, the final stage of spermatogenesis is the morphological shift denoted as spermiogenesis. Here, the sperm cell acquires its characteristic shape, including a species-specific streamlined head containing penetrative enzymes, a structured midpiece, a propelling tail, and the condensation of the male genome [4]. In most cells, nuclear DNA is organized by histone proteins [33]. However, during spermiogenesis, somatic histones are replaced by protamines, the dominant nuclear proteins of mature spermatozoa [33]. The strict compaction of protamine-DNA complexes prevents transcription, provides translational control, and promotes stability in the genome until penetration of the oocyte [33]. This final form produced via spermiogenesis is known as a spermatozoon and is released into the lumen of the seminiferous tubule during the event of spermiation [4,32]. The entire process of spermatogenesis takes approximately 57 days in the stallion [3,7].

Following spermatogenesis, spermatozoa are exposed to a variety of proteinic and non-proteinic substances secreted by the accessory sex glands which aid in the acquisition of mature male fertility and sperm survival during transportation through the male tract and into the female tract [13,14,15,34]. However, the remainder of this review will be focusing on the mature ejaculated spermatozoa and its relation to IVP, a process during which seminal plasma is largely removed, and, thus, we will not be elaborating on the significance of accessory sex glands and their secretions.

## 3. Sperm Morphology

**Figure 2:**
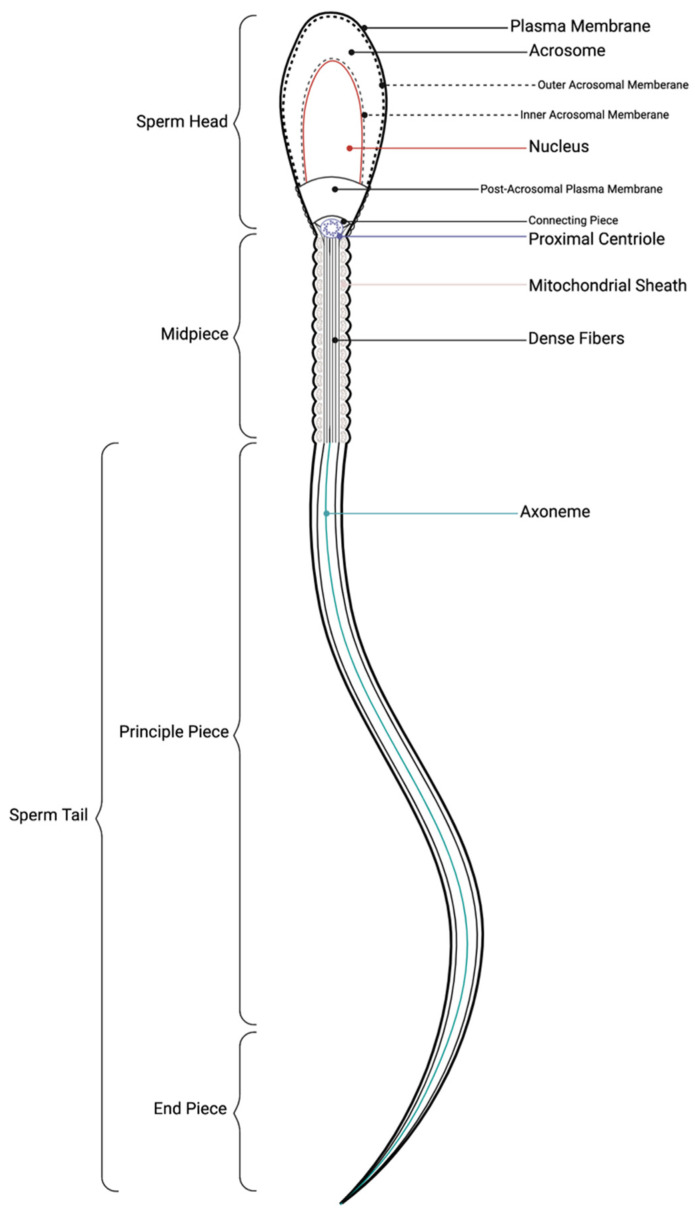
Anatomy of spermatozoa. A spermatozoon consists of three major components: head, midpiece, and tail. The sperm head is overlaid by a plasma membrane, and an acrosomal compartment containing enzymes to aid in fertilization. The nucleus, surrounded by the nuclear envelope, contains the compacted male genome. The head is connected to the midpiece by the connecting piece. The midpiece consists of the proximal centriole, the mitochondrial sheath, and an inner dense fiber structure. The tail points distally, is also covered by a plasma membrane, surrounding the structural axoneme.

The length of the equine spermatozoa from head to tail is approximately 60 µm [35]. A spermatozoa consists of three main components: a headpiece, midpiece, and tail which are fully encapsulated by a plasma membrane (Figure 2) [7]. The sperm head is an elongated, oval shape that is also relatively flat, with some variation on an individual basis [7,36,37]. The head consists of the acrosome, the post-acrosomal lamina, and the nucleus. The acrosome covers the anterior portion of the sperm head and contains hydrolytic enzymes which are released in order for the sperm to penetrate an oocyte [35]. In addition, it is theorized that the proteases and hydrolases contained within the acrosome play a role in the penetration of the oocyte cumulus complex, in addition to the zona pellucida [38,39]. The post-acrosomal lamina covers the caudal nucleus, which contains the highly condensed male genome [7,35]. Species specific traits of the stallion sperm head include a characteristic asymmetrical head, a paraxially inserted tail, and a small acrosome relevant to other species [40].

The neck piece connects the sperm head to the tail and is made up of the connecting piece, the proximal centriole, and mitochondria. The neck serves as a connection point as well as orienting the tail distally [7]. The midpiece is made up of the cytoskeletal axoneme, which contains cylindrically arranged contractile microtubule doublets with attached dynein arms, which serve to facilitate tail movement. Each doublet is surrounded by dense fibers, which are, in turn, surrounded by a double spiral of mitochondria. The mitochondrial helix is critical for supplying energy to the sperm tail, allowing for the motility that is necessary in fertilization events. The end of the midpiece is defined as the caudal end of the mitochondrial sheath where the annulus, a ring of dense filaments, is located to separate the mitochondria and the sperm tail [7].

The principal piece of the propelling tail consists of the continuation of the axoneme and tapering dense fibers. The distinguishing feature of the principal piece is a protein-rich fibrous sheath that provides structure and flexibility for tail movements. The end of the fibrous sheath indicates transition from the principal piece to the end piece, which solely consists of the axoneme. All of these components are covered superficially by the sperm plasma membrane. Although parameters of a morphologically normal sperm may vary significantly on an individual basis, abnormalities in the sperm anatomy may be an indication of subfertility or a problem with spermatogenesis [7].

The outer plasma membrane can be partitioned into the acrosomal, post-acrosomal, neck, midpiece, and principal piece domains [41]. Each region of the membrane can be characterized by a phospholipid bilayer of heterogeneously expressed lipids, proteins, carbohydrates, and cholesterol that is primarily established during spermatogenesis [33,41,42,43]. The cell surface is additionally covered by a glycocalyx, a network of glycoproteins and glycolipids attached to a matrix of oligosaccharides and polysaccharides, that is known to aid in the proper function of sperm, as well as survival as it passes through the female reproductive tract [43,44]. However, spermatozoa in several species, including the ram, bull, rat, boar, buck, man, and stallion have been documented to undergo significant remodeling to the lipid and protein compositions during epididymal maturation [41,45,46,47,48,49,50].

Due to the compaction of the sperm genome and the reduction in transcription, significant changes in protein, lipid, and sugar contents are thought to be a result of the uptake of epididymal epithelial secretions [51]. Although the mechanisms of proteomic alteration are not well understood, several corresponding hypotheses exist, including (a) the reorganization of proteins into membrane specific domains [52], (b) the secretion of soluble proteins into the epididymal lumen by the epithelium and their subsequent absorption and integration into the plasma membrane [52], (c) the release of extracellular vesicles such as epididymosomes and proteasomes from the epididymis contributing micro and transfer RNAs as well as proteins [53,54,55], and (d) the potential direct anchoring of sperm heads to the epididymal epithelium for protein transfers via an unknown mechanism [56]. Specific proteomic changes to the sperm have been associated with various sperm functions including motility (flagellar, signaling cascade, and metabolic modifications) [57,58,59,60], capacitation (uptake of capacitation linked kinases) [61], acrosome reaction (modifications to the scaffolding proteins involved in acrosomal fusion and synapse) [62], and fertilization (facilitation of sperm-zona pellucida and sperm-oocyte interactions) [51,63,64,65,66].

In the stallion, remodeling of the plasma membrane has been partially described through the domain-specific patterning of filipin–sterol complexes acquired during epididymal maturation as well as changes in intermembrane proteins [40]. Changes in protein composition have been thoroughly described in several species, and a majority of studies focus on the acquisition of epididymal secretory proteins between the caput and caudal epididymis [41]. Through freeze-fracture analysis, altered quantities and distributions of various intramembrane particles were observed over the course of epididymal transit in the equine testis, which is hypothesized to play a role in the establishment of various functional domains [50,67]. It is hypothesized that functional domains assist the sperm cell in adapting to new conditions in the seminal plasma and female reproductive tract [41]. Specifically, changes in the binding affinity between sugar-binding lectins and the sperm glycocalyx indicate an altered exposure of terminal saccharide residues in the sperm membrane—thus altering the ability of the sperm to interact with its environment, such as within the uterus and oviduct, or with an oocyte [43,68].

One of the physiological outcomes of membrane protein modifications is the overall change in net surface charge. This characteristic can be estimated through the measurement of zeta potential, or electrophoretic mobility: an electrostatic potential at the slipping plane of the cell [69,70]. The slipping plane can be described as the distance from the cell at which surrounding fluid particles are no longer bonded or attached to the cell, but are completely mobile and free, and the charge at this location is proportional to surface-charge density [71,72]. Zeta potential of sperm cells has been investigated in men, rats, bulls, rabbits, golden hamsters, guinea pigs, and mongoose [69,73,74,75,76]. The source of the net negative charge is due to the addition of negatively charged sialoglycoproteins to the glycocalyx, such as the bipolar glycopeptide CD52, that appear in the sperm membrane during epididymal maturation [43,69,77,78]. These proteins, as well as the total glycoproteic population in the plasma membrane, undergo compositional changes throughout maturation, capacitation, and acrosome reaction, and are thought to play a physiological role in these processes as well as in fertilization [41,77,79]. Thus, membrane charge is both a revealing and complex trait to accurately measure and interpret.

## 4. Bioenergetics and Generation of Motility

As previously mentioned, the mitochondrial helix is the primary grouping of organelles responsible for active motility and metabolism in the sperm cell. The number of mitochondrial gyres in the midpiece of the equine spermatozoa varies between 40 and 50, and their organization, or more specifically a disrupted organization, has been shown to play a role in the fertility of stallions through localized ATP production for sperm flagellar movement [80,81,82]. In fact, mitochondrial function, which can be approximated by mitochondrial membrane potential and electron transport chain (ETC) activity, are known to be positively correlated with overall sperm function [82,83,84,85].

ATP production occurs on the inner mitochondrial membrane within the intermembrane space between inner and outer membranes [6,86]. In the process of oxidative phosphorylation, the primary mechanism of ATP generation in stallion sperm, a mitochondrial membrane potential is established as electrons are passaged through the respiratory enzyme complexes of the ETC of the inner membrane and energy is stored in the form of a proton gradient [82,87,88,89]. Ultimately, ATP synthase uses the proton gradient to generate ATP [6,88]. The mitochondrial membrane potential must be maintained, as reduced polarization can lead to an ATP shortage and cellular damage and hyperpolarization may produce an over-abundance of reactive oxygen species (ROS) and cause lipid peroxidation, which can be detrimental to overall cell integrity [6,90]. It is also noteworthy that oxidative phosphorylation (the primary method of ATP generation in stallion sperm) coupled with mild oxidative stress is beneficial to sperm functional pathways such as hyperactivation, capacitation, acrosome reaction, and fertilization [89]. Lesser amounts of ATP may be produced by glycolysis under oxygen depleted conditions for maintenance of high sperm velocity [91,92]. Additionally, research in stallions has shown correlations between ROS and motility, viability, and mitochondrial function [87,91,93], and, thus, it is highly beneficial to understand mitochondrial mechanisms as they relate to sperm fertility.

## 5. Common Abnormalities and Issues with Fertility

Sperm analysis is a significant method of infertility diagnoses and is critical in order to maximize IVP outcomes. Common issues in patients with male factor infertility can be either obvious or indiscernible to the human eye, and thus the depth of analysis by a technician depends on the technology immediately available to them. Due to the ease of analysis, sperm motility, viability, and morphology are the most common sperm assessments.

Sperm motility is essential for in vivo fertilization and in vitro fertilization (IVF), and is not necessarily required for ICSI where the sperm is manually injected [94,95,96,97]. Sperm motion can be described as either motile or hyperactivated; the latter being a result of capacitation that is required for oocyte penetration. Generally, clinics use Computer Assisted Sperm Analysis (CASA) or similar technologies to reduce subjective errors. CASA can also analyze more complicated motion parameters including the amplitude of lateral head displacement, average path velocity, straight line velocity, curvilinear velocity, linearity of the curvilinear path, and beat-cross frequency [98]. Sperm motility measures are widely considered to be indicative of fertility based on obvious biological functions, despite variable correlations with other sperm quality parameters [97,99]. In the stallion, progressive motility is used as a general estimate of fertility, with less than 50% progressively motile in raw semen or less than 10% progressively motile two hours post collection being an indicator of potential subfertility [100]. However, stallion fertility may be poor even with a highly motile population [101], and, thus, it is critical to understand other common sperm abnormalities.

Sperm viability is a generalized term that can be used to describe a number of traits, including an intact membrane, metabolic activity, and overall physiological health of the cell [102]. Generally, in sperm analysis, viability of the population is estimated by determining the percent of intact membranes using fluorescent dyes such as propidium iodide (PI) and Hoechst 33528 [103,104]. Although Hoechst is permeable with all cells, PI is only able to penetrate cells with disrupted plasma membranes. Thus, staining with two nuclear dyes is necessary for the identification of the non-viable population. Another double staining fluorescent method for viability used in the equine industry is SYBR-14 and PI for viability [103,105]. Assessment of sperm viability can also be indicative of early apoptotic changes, which could also be correlated with other severe sperm abnormalities or infertilities. Rather than, or in addition to, a viability stain with a permeable cell marker, another fluorescent dye may be added to expand upon the assessment of sperm integrity or function. Common fluorescent dyes used for equine sperm assessment include JC-1 [106,107] or rhodamine 123 [108,109] for mitochondrial membrane potential (an estimate of mitochondrial function), fluorescently conjugated Annexin-V (detection of apoptosis) [110,111], or fluorescein isothiocyanate-PNA-Lectin (FITC-PNA) (assessment of acrosomal status) [112,113]. Fluorescent dyes are a common method of sperm quality assessment and a more extensive discussion of their use in ARTs can be found in Section II of this review.

**Figure 3:**
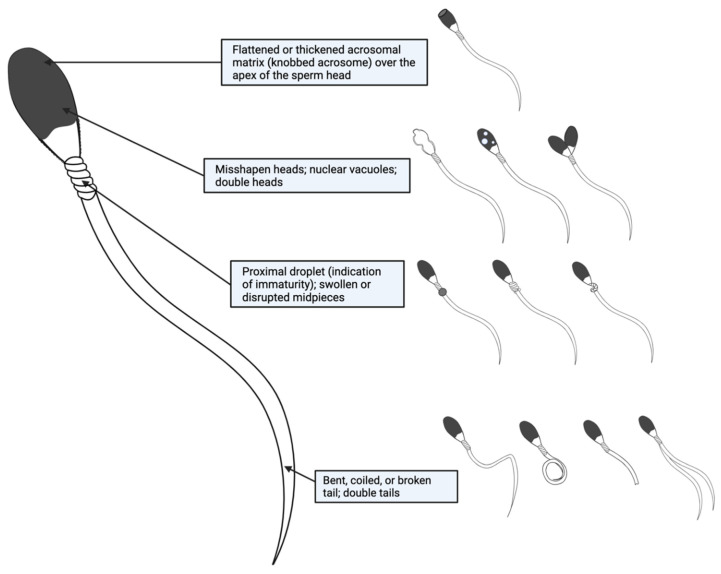
Common abnormalities of equine spermatozoa. Visualizable sperm abnormalities can be broken down by anatomical region: acrosome, head, midpiece, and tail.

Common morphological abnormalities seen in equine spermatozoa may include bent, coiled, or broken tails, misshapen heads, flattened or thickened acrosomal matrices over the apex of the sperm head, nuclear vacuoles, the presence of proximal droplets (an indication of immaturity), swollen or disrupted midpieces, and double heads or tails (Figure 3) [7]. In humans, morphology has been identified as an indicator of quality, and worsened morphology is specifically correlated with poor motility, DNA fragmentation, chromatin immaturity, high levels of ROS, a decreased ability to bind to the oocyte zona pellucida, and an overall decreased fertilization potential [114,115,116,117,118]. Similarly, studies in stallions have identified correlations between morphologic features, motility, and pregnancy outcomes [80,119], indicating that there may be other sperm parameters associated with morphological abnormalities.

Prior to fertilization, the acrosome undergoes a calcium-dependent exocytotic reaction (acrosome reaction) as a result of sperm-oocyte binding that is essential for the subsequent penetration of the oocyte [120]. In equine spermatozoa, the precursor to the acrosome reaction is sperm activation, or capacitation, which occurs in the female reproductive tract as the spermatozoa approaches the oocyte. Capacitation can be generally characterized by the acquisition of both hyperactive motility and the ability to undergo the acrosome reaction through various molecular pathways and protein phosphorylation cascades [51,121,122]. Capacitation has been successfully performed in vitro in numerous species, including humans and horses [122]. The acrosome reaction has also been achieved in vitro for the horse by using various components, including calcium (Ca^2+^), calcium ionophore, bicarbonate (HCO_3_^−^), lysophosphatidylcholine, and progesterone leading to calcium oscillations [11,112,122,123,124]. Interestingly, sperm from stallions classified as fertile based on their breeding history are more likely to undergo the acrosome reaction in vitro when incubated with progesterone than sperm from subfertile stallions [125]. In humans, in vivo-derived inducers of calcium oscillations leading to the acrosome reaction include follicular fluid, cumulus oophorus, and the presence of granulosa cells; however, these methods are not well understood in the horse [122,126,127,128,129].

In the context of fertilization, capacitation involves calcium oscillations that trigger a complex cascade of intracellular events leading to the binding of specific zona ligands on the outer plasma membrane with the zona pellucida of the oocyte [130,131]. Subsequently, the acrosome reaction, or the fusion of the outer acrosomal membrane with the sperm plasma membrane, is marked by the exocytosis of proteolytic and hydrolytic enzymes from the acrosomal compartment [132,133]. These enzymes aid in the digestion of the zona pellucida so the sperm can penetrate the zona pellucida using hyperactivated motility acquired during capacitation. This results in the entrance of the sperm into the perivitelline space and the fusion of the inner acrosomal membrane and the equatorial region of the sperm head with the oolemma [133,134,135]. However, if a sperm cell undergoes the acrosome reaction prematurely, which can occur during cryopreservation or in vitro processing, it loses its ability to penetrate the cumulus oophorus and zona pellucida for fertilization [136,137]. In human in vitro experiments, a premature acrosome reaction precluded the binding of sperm to the oocyte, and sperm that were able to bind were less successful in penetration [131,138]. In horses, it has been demonstrated that sperm from subfertile stallions bind less frequently to the zona pellucida of the oocyte than sperm from fertile stallions, and that sperm from subfertile stallions is less likely to undergo acrosome reaction after binding [139], indicating discrepancies between fertile and subfertile sperm membrane affinities and compositions. Therefore, it is of interest to remove the prematurely acrosome reacted spermatozoa during selection procedures.

The mitochondrial helix is a sensitive structure that can be easily damaged under extreme environmental conditions, including cryopreservation [6]. Disruption of mitochondrial integrity, including the depolarization of the membrane, can disrupt ATP production and cause a sperm cell to become immotile and non-functional [6,90]. Alternatively, hyperpolarization of the mitochondrial membrane will lead to lipid peroxidation and an over-abundance of ROS, leading to cellular damage [6,90]. Although exact mechanisms of cryoinjury to equine sperm are poorly understood, potential targets include disrupted plasma and mitochondrial membranes, increased ROS production, and generation of apoptotic factors [6,93,140].

Apoptosis is also a common issue seen in sperm samples, especially those that undergo thermal, oxidative, or osmotic stressors from extending, cooling, or cryopreservation [141,142]. These stressors, as well as abnormal morphology, can initiate a variety of negative effects such as membrane and mitochondrial damage, plasma membrane restructuring (including the externalization of proteins such as phosphatidylserine), generation of ROS, and subsequent DNA damage [10,12,141,142,143].

DNA integrity assessment is one of the most valuable assessments of sperm fertilization potential due to the strong correlation with sperm reproductive competence; in fertilization as well as in subsequent embryo development and offspring phenotype [144]. Poor DNA integrity of sperm, or sperm with increased DNA fragmentation, can, thus, have detrimental effects on reproductive outcomes. DNA fragmentation is an all-encompassing term that includes both single- and double-stranded breaks, single base deletions or modifications, various non-desirable cross linkages, and mispackaging errors [145]. Causes of DNA fragmentation may include the mispackaging of chromatin during spermatogenesis [146], apoptosis [147], excessive ROS [146,148], and other environmental factors [144]. The use of spermatozoa with damaged DNA has been associated with compromised fertilization both in vivo and in vitro, as well as negative effects on embryo development, such as worsened embryo quality and blastocyst rates [144,149]. This could potentially lead to both miscarriages and altered offspring phenotypes including genetic diseases, such as Apert syndrome or achondroplasia, conditions thought to arise due to replication error mutations and cancers [144,150,151,152,153]. Thus, DNA integrity of semen can be a good indication of fertilization potential and the potential effects on embryo development and offspring characteristics.

Surface composition and the resulting membrane charge are also of interest in sperm fertility studies. A greater net negative zeta potential, a parameter determined by surface composition as described previously, is acquired during epididymal maturation through extensive membrane remodeling and has been correlated with sperm quality in men [154,155]. The acquisition of a net negative charge is primarily based on the extrusion of sialic acid (sialoglycoproteins) and other charged proteins to the outer membrane of the head region during epididymal maturation [44,69,77,156]. The charge may also change significantly as a sperm changes environments, or when it undergoes capacitation or acrosome reaction [41,79]. Specifically, membrane charge increases, or becomes less negative, when the sperm undergoes capacitation [157]. Externalization of sialoglycans by the sperm has been shown to play a role in avoidance of the uterine immune systems, as well as playing roles in capacitation and being an important component of sperm-zona pellucida binding, and, therefore, fertilization. Thus, charge is a significant factor in sperm fertility [44,77,158,159]. Extrapolating from these data, selecting sperm with a greater net negative zeta potential will theoretically select for mature, functional, and viable spermatozoa.

## 6. Conclusions

Thorough interpretation of sperm physiology, despite its complexity, is the best method for assessing male fertility. In particular, furthering our understanding of the relationships between sperm morphology, viability, biological composition, and metabolism for equine sperm will be extremely beneficial in understanding fertility in stallions, as well as shedding light on associated mechanisms. In addition, characterization of new biophysical properties, such as zeta potential, will not only aid in our understanding of what makes a fertile sperm, but will also allow for the development of new semen selection technologies. For a review of current and prospective sperm selection technologies, please refer to Section II of this review. In conclusion, sperm physiological assessment is an invaluable tool for the equine breeding industry and merits continued consideration in clinical and research settings.

## Data Availability

Not applicable.
